# Effects of 7-Week Hip Thrust Versus Back Squat Resistance Training on Performance in Adolescent Female Soccer Players

**DOI:** 10.3390/sports7040080

**Published:** 2019-04-03

**Authors:** Jaime González-García, Esther Morencos, Carlos Balsalobre-Fernández, Ángel Cuéllar-Rayo, Blanca Romero-Moraleda

**Affiliations:** 1 Education and Health Faculty, Camilo José Cela University, 28692 Madrid, Spain; angelc.rayo@gmail.com (A.C.-R.); bromero@ucjc.edu (B.R.-M.); 2 Exercise and Sport Sciences, Education and Humanities Faculty, Francisco de Vitoria University, UFV, Bulding E, Ctra. M-515 Pozuelo-Majadahonda Km 1,800, 28223, Pozuelo de Alarcón, 28223 Madrid, Spain; esther.morencos@ufv.es; 3 LFE Research Group, Technical University of Madrid, 28040 Madrid, Spain; carlos.balsalobre@icloud.com; 4 Department of Physical Education, Sport and Human Movement, Universidad Autónoma de Madrid, 28049 Madrid, Spain

**Keywords:** countermovent jump, mean concentric velocity, strength, female soccer, force vector theory, transference

## Abstract

Hip thrust (HT) is a loaded bridging exercise that requires more hip extension than a back squat (SQ) does, while in a back squat, triple flex extension occurs. Due to the specificity of each exercise, it is claimed that HT gains can be better transferred to actions where hip extension occurs. In addition, strength improvements during squatting can be transferred in a greater way to vertical plane movement, such as vertical jumping. However, its effects on the performance of female soccer players are unclear. For this reason, the purpose of this study was to analyze a 7-week training program on performance variables using either HT or SQ exercises in female adolescent soccer players without lifting experience (*N* = 24, age = 16.82 ± 1.56 years, height = 1.64 ± 0.55 cm, body mass = 58.35 ± 6.28 kg). Players were randomized into three groups: A back squat group (SQG; *N* = 8), hip thrust group (HTG; *N* = 8), and control group (CG; *N* = 8). Participants in the HTG and SQG joined a progressive resistance training program twice per week for 7 weeks with either HT or SQ exercises. A countermovement jump, 10–20 m sprint, T-test, and barbell velocity during HTs and SQs (with the load that represents ~60 and ~80% RM) were measured before and after the intervention. The HTG showed greater improvements in the 10-m sprint (*d* = 0.7), 20-m sprint (*d* = 0.46), T-test (*d* = 0.36), and barbell velocity at 80% repetition maximal (RM) (*d* = 0.53) and 60% RM (*d* = 1.02) during hip thrusts, while the SQG showed higher barbell velocity at 80% RM (*d* = −0.7) during back squats. These results may be useful for strength and conditioning coaches working with adolescent female soccer athletes, since both strengthening exercises improved performance in different ways due to the nature of the exercise.

## 1. Introduction

Decisive situations during soccer require strength, power, and speed. In particular, straight sprinting is the most determining action in a match result, as it is the most common action that precedes goal scoring [1]. Previous evidence has shown that resistance training has a key role in improving physical capabilities: Increased strength, muscle power, rate of force development (RFD), and the capacity to perform sport demands [2]. However, strength gains may be optimized according to load vector orientation [3,4,5,6,7]. 

Hip thrust (HT) and back squat (SQ) are two exercises used to improve lower limb strength [8,9,10,11,12,13]. Hip thrust is a horizontally loaded bridging exercise that requires hip extension moment production to displace a load [14]. Because of this exercise’s horizontal nature, it has been theorized that it may be great transferred to sports in which hip extension occurs and thus horizontal force production is needed [15].

On the other hand, the back squat is an axially loaded exercise in which triple flex extension (hip, knee, and ankle) occurs [16]. Despite the close relationship between back squat and horizontal movements (i.e., sprinting) [17] from an electromyographical point of view, there is greater electromyography (EMG) activation of hip extensor muscles in hip thrust than in back squat for the same relative load (10 RM) [18]. In addition, there is research that has attempted to identify the mechanical variables of linear sprint performance. Much of the research has shown that horizontal ground reaction force (GRF) correlates with maximal speed, mean speed, and the distance covered in 4 s during the sprint. In comparison, vertical GRF only correlates with top speed [19]. Due to the importance of sprinting and acceleration in team sports, some research has analyzed the influence of strength training programs in those performance variables, showing that improvements have vector-specific transference [8,9,10,20,21]. 

To the best of our knowledge, there is only one study that has investigated differential benefits between barbell hip thrusts and front squat exercises [10]. However, it was only conducted with male rugby participants. The study observed that in the hip thrust group, there were greater improvements in sprint, horizontal jump, and hip thrust 3 RM. In comparison, there were greater improvements in the vertical jump and front squat 3 RM for the front squat group. While these results were promising, there is currently no existing research that has compared the effects of hip thrust exercises to back squat exercises, especially in adolescent female soccer players. As the training background modifies the relationship between force–power–velocity variables and performance parameters [22], the purpose of this study was to compare performance differences between hip thrust and back squat exercises in a 7-week training program.

## 2. Materials and Methods

### 2.1. Study Design

This study compared strength adaptations, running, and changes in speed direction in response to a 7-week training program of hip thrust or back squat resistance training. The study took place at the end of the competitive season, with players being given two complete rest days before the test. Players were instructed to refrain from physical activity during rest days. This was a single-center, parallel-group, randomized controlled trial with equal randomization (1:1). Each group was assigned to perform the hip thrust or back squat twice per week for 7 weeks for a total of 14 sessions and 48 h of rest between sessions. Performance variables were collected before and after the 7-week training period.

### 2.2. Participants

Twenty-four adolescent female soccer players (age: 16.82 years ± 1.56; height: 164.3 cm ± 5.52; body mass: 58.35 kg ± 6.28, without previous lifting experience) participated in the study and were randomized into three groups: A back squat group (SQG, *n* = 8), hip thrust group (HTG, *n* = 8), and control group (CG, *n* = 8). No injuries were reported at the starting point of the training protocol. All participants were enrolled in a female soccer team (national sub-elite level, three training sessions/week). 

During the intervention period, participants were not allowed to perform lower limb strength training outside of the experiment protocol. To ensure optimal performance and the safety of all participants, two orientation sessions were conducted. All subjects were asked to abstain from unaccustomed exercise along with any medications and/or dietary supplements. Written informed consent was obtained from all subjects and their legal guardians: Moreover, the researchers met with the participants to explain the study design and the requirements. The study and informed consent procedures were approved by the Camilo José Cela Ethics Committee in accordance with the latest version of the Declaration of Helsinki. Prior to pre-intervention assessment, subjects participated in a familiarization session. During this session, all participants performed every test and were technically evaluated in both strengthening exercises [14,16]. Another aim of this session was to teach athletes to lift with maximal intentional velocity.

### 2.3. Procedures

**Figure 1:**
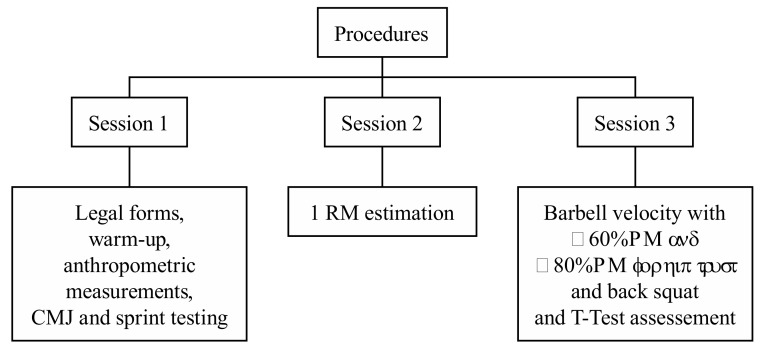
Baseline assessment schedule.

Pre-intervention evaluations took place during four sessions in the same week. During the first session, subjects completed the legal forms and baseline measurements were taken, including a countermovement jump (CMJ), 10-m sprint, and 20-m sprint following a warm-up routine (Figure 1).

During the second session, 1-repetition maximal (RM) back squats and hip thrusts were measured. Prior to the 1-RM estimation, participants were re-evaluated for their technical execution of both exercises [14,16]. Four lifts with moderate to high loads were performed to estimate the 1-RM. In the third session, mean concentric velocity in both exercises was measured through accelerometry. Participants performed two repetitions with two different loads (60% RM and 80% RM for hip thrusts and back squats), with maximum intentional velocity during lifting. During the same session, a T-test was conducted [23]. Before evaluations, a warm-up routine was performed during each of the four sessions. In each of the four sessions, a specific warm-up was performed. 

The specific warm-up routine was an adaptation of the “10-minute lower body dynamic warm-up”, as previously described by Contreras et al. [10]. This warm-up consisted of 5 min of jogging followed by 10 repetitions of these exercises: leg swing sagittal plane, leg swing frontal plane, deep body load squat, and body load hip thrust. Any further references to the specific warm-up refer to this procedure.

Anthropometric measurements were carried out following the International Society for the Advancement of Kinanthropometry (ISAK) six skinfold footsteps [24]. All measurements were performed twice by two different anthropometrists. Triceps, subscapulars, suprailiacs, abdominals, thighs, and medial leg skinfolds were measured with calibrated calipers. The perimeters of the arms, thighs, and legs were also measured.

For the vertical jump assessment, the My Jump 2 iOSApp, installed on an iPhone 6 running iOS 11.1.1, was used. This application calculates a jump’s flight time by identifying the take-off and landing frames with the help of the iOS device’s high-speed camera. The jump height was calculated by the following equation: *h* = *t^2^* × 1.22625, with *h* being the jump height in meters and *t* being the flight time of the jump in seconds [25]. Three CMJs without arms were assessed, with 3 min of rest between each attempt. Subjects were requested to maintain their hands on their hips during the jump. Knee flexion was not allowed during the flight phase. If any of these parameters were not followed, the trial was repeated. As previously assessed and to ensure maximal jumping height, the best of three jumps was used for statistical analysis [3,5,6,7]. Power (W), force (F0), and take-off velocity (V0) were also calculated, according to Samozino´s equations [26]. All subjects were experienced with this test. All of them had performed, at least, four CMJs previously.

Running speed was evaluated by 20-m sprint times (0–20 m), with 10-m (0–10 m) and 20-m (10–20 m) split times using three sets of single-beam timing lights (DSD Sport system, León, Spain). The starting line was placed 50 cm before the first timing gate. To ensure the correct performance of the speed test, subjects performed 20 m of a submaximal sprint at 70%, 80%, and 90% of their subjective maximal velocity prior to data acquisition. After approximations with submaximal velocities, subjects performed three trials at maximum velocity. The best value of the three attempts was taken for statistical analysis.

For the change of direction assessment, subjects performed the T-test. This test consists of placing four cones in a T formation. The two central cones were separated by 10 m, and the central front cone was separated laterally from the left one by 5 m, the same distance as the right one [23]. All subjects performed the test twice, with 5 min of rest between each attempt. The best result of both attempts was used for statistical analysis. Time was collected using one set of single-beam timing lights (DSD Sport system, León, Spain) situated at the starting/finish line of the test.

During the second and third day of the baseline assessment, the 1-RM was estimated through My Lift, a validated iOS App installed on an iPhone 6 running iOS 11.1.1 [27]. This app was designed to measure barbell velocity through slow motion video recording of a lift. The app allowed a frame-by-frame analysis of the video in order to manually select the beginning and end of the lift. Previous range of movement (ROM) was measured. Finally, mean vertical barbell velocity (m·s^−1^) was computed by dividing ROM by the lift time. Back squat ROM was calculated as the vertical difference between the height of the barbell with respect to the ground at the final position and the height of the barbell at the bottom position. Hip thrust ROM was assessed through a similar method: It was calculated as the vertical distance between the ground and the bottom of the plate during the final position. The first frame in which the plate ascended (in a back squat) or left the ground (in a hip thrust) was considered to be the beginning of the concentric phase. The end was considered to be when there was no more vertical displacement of the plate. The application is also designed to estimate the 1-RM using the force (load)–velocity relationship [28]. To estimate the 1-RM, the application’s algorithm needed four different submaximal loads and each load’s corresponding barbell velocity and 1-RM velocity (*v*-1-RM). Using these variables, an individual load velocity profile was created using least squares to obtain a linear regression equation: v=sm+i, where v is the velocity (in m·s^−11^), m is the mass of the system (kg), s is the slope of the straight line equation, and i is the *y* intercept. Thus, the mass of the system can be calculated as $m=\frac{v-i}{s}$, as v is the value of the velocity associated with the 1-RM [28,29]. To ensure the correct 1-RM estimation, participants were instructed to perform the concentric phase of the lift as fast as possible.

During session four, mean concentric velocity in both exercises, with 80% RM and 60% RM, was measured. For both exercises, this assessment was conducted with the Push™ accelerometer (PUSH Inc., Toronto, Canada). This wearable device calculates vertical mean concentric velocity by integrating vertical acceleration with respect to time. The PUSH™ band then calculates the mean velocity of the movement by averaging all instantaneous velocities registered during the concentric phase. The wearable device revealed a very high association with linear transductor (LT) mean velocity (*r* = 0.86, standard error of the estimate = 0.08 m·s^−1^). The PUSH™ band also showed a high level of agreement with LT (Intraclass Correlation Coefficient = 0.907) [30]. To record the measured data with the PUSH™ band, the system was linked to an iPhone PUSH™ app v.1.10.4 (PUSH Inc., Toronto, Canada), using a Bluetooth™ 4.0 LE 200Hz recording rate (Bluetooth Special Interest Group Inc., Bellevue, WA, USA), connection. Subjects were asked to perform two lifts as fast as possible. For statistical analysis, mean concentric velocity (m/s) and mean power (W) were used during the lifts of both exercises.

After baseline assessment, participants were randomly assigned to one of the three groups (HTG, SQG, or CG). The HTG performed hip thrusts during the intervention period, the SQG performed back squats, and the CG did not perform any strength training. The intervention groups continued training with their respective teams.

The exercises’ intensities started with 60% of their estimated 1-RM. The load was gradually increased over the 7-week intervention, up until 90% of the 1-RM. All workouts were separated by, at least, 48 h [18]. Prior to each session, the specific warm-up was performed. The training schedule is shown in Table 1. Three minutes of rest was taken between sets, and subjects were requested to move the load as fast as possible in all repetitions.

At the end of the intervention period, post-data were collected during week 8. The minimum adherence requirement to complete the intervention period was established as one completing 85% of planned workouts. Of the 24 subjects who began the intervention, a total of 22 successfully completed the intervention. The two missing subjects were eliminated due to the lack of adherence.

### 2.4. Statistical Analysis

Data are presented as mean ± standard deviation. All data were entered into the statistical analysis program SPSS Statistics 21 (SPSS Inc., Chicago, IL, USA), and Shapiro–Wilk tests were performed to ensure normality. Values of *p* ≤ 0.05 were indicative that these values had a normal distribution. Effect size (ES) statistical analysis was calculated using Cohen‘s *d* ((between groups: $d=\frac{M1-M2}{SD\;pooled}$, where *M*1 and *M*2 are the mean changes (*Mpost*–*Mpre*) for each group, and *SDpooled* is the pooled standard deviation of changes from each group. Within groups, $d=\frac{Md}{Sd}$, where *M_d_* is the mean difference from pre-to-post and *S_d_* is the standard deviation of differences between subjects, which was defined as small, medium, and large for 0.20, 0.50, and 0.80, respectively [31]. Quantitative chances of beneficial/better or detrimental/poorer effects were assessed qualitatively as follows: 0–5% very unlikely, 5–25% unlikely, 25–75% possible (unclear within group assessment), 75–95% likely, and 95–100% very likely [32]. A substantial effect was set at >75%. If the chances of having beneficial/better and detrimental/poorer performances were both >5%, the true difference was assessed as unclear. Ninety percent (90%) confidence limits (CLs) of the ES were also calculated.

## 3. Results

Table 2 shows a description of the sample in the baseline assessment.

**Figure 2:**
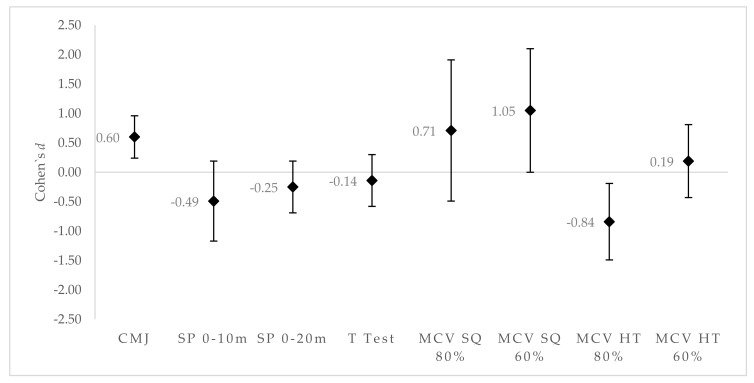
Differences in effect size (ES): Back squat group versus control group. Positive values favor SQG. Negative values favor CG.

The SQG showed greater effects in CMJ performance (*d* = 0.6 [0.24;0.96], with chances for greater/similar/lower CMJ in comparison to CG of 96%/3%/0% medium/very likely) and mean concentric velocity in the back squat with 60% RM (*d* = 1.05 [0.01;2.01], with chances for greater/similar/lower mean concentric velocity in comparison to CG of 91%/6%/3% high/likely), while the other variables did not improve performance regarding the CG (Figure 2).

**Figure 3:**
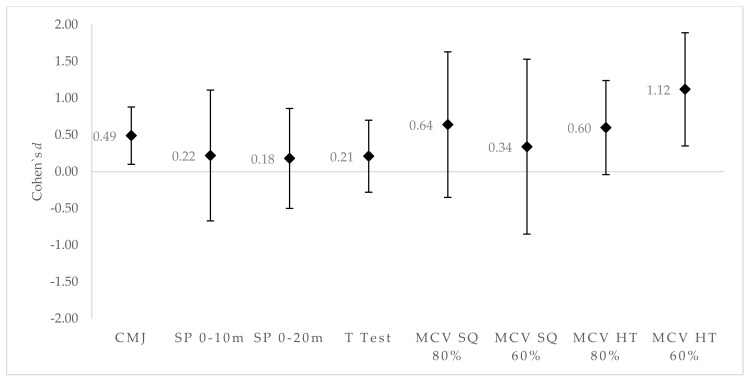
Differences in ES: Hip thrust group versus control group. Positive values favor HTG. Negative values favor CG.

The HTG showed greater effects on CMJ performance (*d* = 0.49 [0.1;0.88], with chances for greater/similar/lower CMJ in comparison to CG of 90%/10%/0% small/likely) and mean concentric velocity in the hip thrust with 60% RM (*d* = 1.12 [0.35;1.88], with chances for greater/similar/lower mean concentric velocity in comparison to CG of 97%/2%/1% high/very likely) (Figure 3). Trivial to small effects on performance were seen for the sprint 0–10 m (*d* = 0.22 [−0.67;1.12], with chances for greater/similar/lower mean concentric velocity in comparison to CG of 52%/28%/20% small/unclear), sprint 0–20 m (*d* = 0.18 [−0.5;0.86], with chances for greater/similar/lower mean concentric velocity in comparison to CG of 48%/36%/17% trivial/unclear), and T-test (*d* = 0.21 [−0.28;0.7], with chances for greater/similar/lower mean concentric velocity in comparison to CG of 52%/40%/8% small/unclear).

The ES results between the SQG and HTG are shown in Table 3. The HTG produced substantially better results in the 0–10 m sprint (*d* = 0.7 [−0.16;1.56], with chances for lower/similar/greater sprint time in comparison to SQG of 85%/11%/4% medium/likely), sprint 0–20 m (*d* = 0.46 [−0.2;1.13], with chances for lower/similar/greater 20 m sprint time in comparison to SQG of 76%/19%/5% small/likely), T-test (*d* = 0.36 [0.08;0.65], with chances for lower/similar/greater T-test time in comparison to SQG of 84%/16%/0% small/likely), and hip thrust concentric velocity with 80% RM (*d* = 0.53 [−0.2;1.27], with chances for greater/similar/lower mean concentric velocity in comparison to SQG of 78%/17%/5% medium/likely) and 60% RM (*d* = 1.02 [0.13;1.91], with chances for greater/similar/lower mean concentric velocity in comparison to SQG of 94%/5%/2% high/likely).

The SQG showed greater effects in back squat mean concentric velocity with 60% RM (*d* = −0.7 [−1.59;0.18], with chances for greater/similar/lower mean concentric velocity in comparison to HTG of 84%/12%/5% medium/likely). 

It is unlikely that one intervention was better than the other for improving CMJ (*d* = −0.02 [−0.36;0.31], with chances for greater/similar/lower mean concentric velocity in comparison to SQG of 13%/69%/18% small/unclear) and back squat mean concentric velocity with 80% RM (*d* = −0.03 [−0.7;0.64], with chances for greater/similar/lower mean concentric velocity in comparison to SQG of 27%/41%/32% trivial/unclear).

## 4. Discussion

This study was designed to compare the performance effects of a 7-week training program, utilizing either hip thrust or back squat exercises, in adolescent female soccer players without lifting experience. Our main findings revealed small and unclear improvements in sprint times and T-tests for the HTG. However, there were medium to large improvements in hip thrust mean concentric velocity (MCV) with both loads. In addition, the SQG showed medium to large effects on performance in the CMJ and MCV with both loads. These results agreed with the principle of specificity, since these variables had the same external force vector direction [33]. In addition, eight weeks of a back squat training program with loads ranging from 40% to 60% of the 1-RM showed potential beneficial effects on vertical jump height (Δ = 6.4 [3.5;9.2] 99%/1%/0%) [11]. These results were similar to ours: SQG training showed very likely beneficial effects for CMJ height (Δ = 10.4 [4.1;17.2] 97%/3%/0%). Hip thrust training caused potentially beneficial effects on CMJ performance (Δ = 9.9 [1.9;18.5] 90%/10%/0%) regarding the CG. Recent research has provided similar information, with 14-week hip thrust training increasing vertical jump by ~6% [12]. Therefore, when both groups were compared, unclear effects between intervention groups for CMJ height were observed (Δ = −0.5; 13%/69%/18%; Table 3). This might be explained by the importance of hip extension in vertical jump performance. Previous research has shown that vertical jump improvements had a notably larger association with concentric peak hip power (*r* = 0.61) than concentric peak knee and ankle power (*r* = 0.33; *r* = 0.32) [34]. Therefore, exercises emphasizing concentric hip power extension (e.g., a hip thrust) could improve vertical jump performance in a way similar to exercises in which hip, knee, and ankle extensions occur (e.g., a back squat). However, our results differed from those obtained by Contreras et al. (2017) [10]. In this investigation, the front squat group showed higher potential beneficial effects in vertical jump height (*d* = −0.47 [−1.20;0.23]). 

It is well known that positive transfer occurs between increases in lower limb strength parameters and sprint performance [17]. This relationship has traditionally been explicated by increases in peak resultant ground reaction force (pGRF) and rate of force development (RFD) during sprint running [35]. However, a previous approach has demonstrated that the principal determinant of straight sprint speed and straight sprint acceleration was the horizontal ground reaction force (hGRF), while the vertical ground reaction force (vGRF) only correlated with maximal speed [19]. With this suggested approach, recent research has shown that six to eight weeks of anteroposterior (e.g., hip thrust) or vertically oriented (e.g., back squat) training could either potentially modify [10,11,13] or not modify 0–10 m and 0–20 m sprint performance [9]. Only one of those investigations compared the effects of both resistance exercises on sprint performance [10]. What was observed was that the hip thrust group showed greater results than the front squat group in 0–10-m and 0–20-m sprint performance (*d* = 0.32; *d* = 0.39). These results agreed with ours (Δ = 5.6, 85%/11%/4%; Δ = 3.1, 76%/19%/5%). However, if we compare the HTG results regarding the CG, our data showed a small and unclear effect on sprint performance (*d* = 0.22 [−0.67;1.12] 52%/28%/20%; *d* = 0.18 [−0.50;0.86] 48%/36%/17%) (Figure 3). The moderate ES between HTG and SQG could have been more influenced due to the loss of performance in the SQG than improvements in the HTG. This detriment in SQG performance did not agree with previous research [17] and may be explained due to the limitations of our study. Training load was not controlled during training sessions, which might have produced different fatigue recovery patterns. It is possible that our participants’ physical functions were influenced during data collection.

Our data also showed that the hip thrust group obtained greater sprint performance in the first split (0–10 m) than the SQG (*d* = 0.7 [−0.16;1.56] 85%/11%/4%) did. These results could be explained due to the importance of hip extension muscles during the first strides of sprint acceleration. Morin et al. [36] have shown that horizontal force (the strongest predictor of sprint and acceleration performance) was significantly related to gluteus maximus concentric torque and electromyographic activity during the first 10 steps of a sprint. Therefore, if hip extensor muscles were targeted using hip thrusts [18], hip extension torque could be increased and transferred to acceleration performance. However, further research is needed: not all investigations have shown beneficial effects in sprint times following hip thrust resistance training [8,9]. Moreover, methodological variations in sprint distance are needed due to the relationship of vGRF and maximal sprint distance, which is reached at ~40 m. Due to the different results obtained from investigations, more data are necessary in order to clarify the effects of horizontal force training on sprint performance.

Changing directions during physical activity is considered to be a multifactorial task. It depends on multiple variables to explain its performance, which include technique, speed, an athlete’s muscular capacities (i.e., reactive force, concentric strength and power, muscular imbalances, and values of eccentric and isometric strength), braking forces, and propulsion impulses [37,38].To date, only one study has shown the importance of lower limb power (measured as CMJ related variables) on change of direction (COD) performance in female soccer players [39]. Researchers have shown that CMJ height and COD performance correlate with each other (*p* = 0.003). However, our data did not show potential benefits in the COD test (*d* = 0.21, 52%/40%/8% for the HTG; *d* = −0.14 10%/40%/50% for the SQG) despite both groups having increased their CMJ height (*d* = 0.49 90%/10%/0% for the HTG; *d* = 0.6 93%/7%/0% for the SQG). To the best of our knowledge, our research is the only one to have observed the effects of an HT training program and its influence on the direction change test. Therefore, we could only compare our SQ group results (*d* = −0.14 10%/40%/50%), which were similar to results obtained in previous research [40]. In that study, eight weeks of strength training with back squats with loads ranging from ~40% to 60% 1-RM did not improve COD performance (*d* = 0.15 46%/31%/23%). The HTG had greater improvements in the T-test. It can be theorized that propulsive impulses are more decisive than deceleration impulses during direction change maneuvers. Only one study has used hip extension strength exercises in their intervention program [7]. They observed beneficial effects in the COD test (*d* = 0.44 98%/2%/0%). However, these effects may have been due to the program’s eccentric overload. Therefore, more research with traditional hip extension strength exercises is needed.

The intervention groups obtained greater improvements in the evaluation of the MCV in the respective exercise they had trained for during the intervention period. That is, the HT group had greater improvements in the MCV test for the hip thrust at 60% RM [Δ = 22.0% (2.5;45.2) 94%/5%/2%] and 80% RM [Δ = 20.0% (6.8;54.4) 78%/17%/5%], while the SQ group had greater improvements in back squat MCV at 60% RM [Δ = −11.7% (−24.4;3.2) 5%/12%/84%]. The role of movement velocity to control effort intensity during resistance training is well known. There is also a strong relationship between movement velocity and relative load (% RM), represented by the external load (kg) [28]. In this research, it was observed that increments of 0.07–0.09 m/s for the same external load could improve 1-RM by 5%. Therefore, if subjects were able to displace the same external load at a higher speed, the relative load that represented the external load decreased. A comparison of both experimental groups showed that the principle of training specificity was fulfilled. 

However, back squat MCV at 80% RM resulted in uncertain results [Δ = −1.0% (−21.0;23.9) 27%/41%/32%]. These results suggested that improvements in hip extension strength could be transferred to back squat performance. Research has shown that as the %RM increased during back squats, the relative muscular effort (the ratio of a muscle group’s net joint moment during a task relative to the muscle group´s net joint moment during the maximal voluntary isometric contraction) of the hip extension increased during different squat depths [41]. 

To the best of our knowledge, this is the first study that has analyzed the effects of hip thrust versus back squat exercises in female soccer players. These results may have potential applications for strength and conditioning coaches working with adolescent female soccer players. However, this study did have a few limitations. First, our subjects were young soccer players without any lifting experience. They may not have had the ability to move the barbell with maximal intentional velocity. As a result, our results regarding barbell velocity and power should be treated with caution. In addition, the CG only carried out the strengthening exercises during familiarization and data acquisition. Another limiting factor was the impossibility of monitoring training load with barbell velocity displacement. If the RM varied daily and movement velocity was not monitored, the proposed external load (kg) based on %RM would not be adequate during the training protocol.

Finally, our results may have been modified by factors that were not considered in this research. For example, training sessions were not planned according to menstrual cycle phases. Recent research has shown that strength training that was performed by subjects during the follicular phase resulted in greater gains than in subjects that trained during the luteal phase [42]. It could also be interesting to obtain kinetic variables associated with sprinting, such as the ratio of forces (RF) or the rate of decrease in the RF with increasing sprint during sprint acceleration (D_RF_) [43], and with how back squats and hip thrusts can modify vertical and horizontal force–velocity profiles (even though the effects of an individualized force–velocity intervention have already been studied [44]). 

## 5. Conclusions

A 7-week resistance training program involving hip thrust or back squat exercises improved performance in adolescent female subjects without lifting experience. Greater improvements in hip thrust MCV with moderate and heavy loads were observed for the hip thrust training group. The back squat resistance training group had greater improvements in back squat MCV with moderate loads. It is unclear if both exercises produced greater improvements for vertical jump and back squat MCV performance with heavy loads. Our results suggest that there is not clear transference between these exercises and the first meters of a sprint and change of direction performance.

## Figures and Tables

**Table 1:** Training schedule. RM: Repetition maximal.

Week 1	Week 2	Week 3	Week 4	Week 5	Week 6	Week 7
4 × 12 (60% RM)	4 × 10 (70% RM)	4 × 10 (70% RM)	4 × 8 (80% RM)	4 × 8 (80% RM)	4 × 6 (85% RM)	4 × 4 (90% RM)

**Table 2:** Baseline assessment variables.

Variables	Squat Group (*n* = 8)		Hip Thrust Group (*n* = 8)		Control Group (*n* = 8)	
–	Mean	SD (±)	Mean	SD (±)	Mean	SD (±)
Mass	58.21	3.83	59.77	7.37	54.686	6.19
Fat%	18.71	3.34	18.5	3.12	17.514	4.41
Musc%	25.2	3.78	24.54	2.57	23	1.68
CMJ	28.96	4.03	25.53	4.9	28.103	3.73
Pow CMJ	1342.11	209.64	1297.33	425.72	1348.75	237.14
F0	1139	131.39	1148.31	281.52	1121.97	163.32
V0	1.18	0.1	1.11	0.11	1.2	0.06
SP 0–10 m	1776.29	73.36	1944.29	92.86	1941.71	149.53
SP 10–20 m	1390.43	49.8	1451.71	81.42	1453.14	102.9
SP 0–20 m	3166.71	96.92	3396.00	135.36	3394.85	246.12
T-test	10.54	0.26	10.95	0.37	11.04	0.53
MCV SQ 80%	0.52	0.17	0.49	0.15	0.48	0.08
MCV SQ 60%	0.68	0.11	0.64	0.1	0.66	0.1
MCV HT 80%	0.63	0.19	0.6	0.16	0.51	0.15
MCV HT 60%	0.73	0.1	0.72	0.14	0.63	0.1
Pow SQ 80%	633.71	238.21	594.71	192.62	628.14	86.32
Pow SQ 60%	745.43	204.03	623.57	84.37	652.71	108.97
Pow HT 80%	467.43	139.21	507.5	199.91	372.43	103.83
Pow HT 60%	418.86	101.01	433	83.59	375.71	63.09

Mass (kg); fat%: Body fat percentage; musc%: Body muscle mass percentage; CMJ (cm): Countermovement jump; pow CMJ (w): Power in countermovement jump assessment; F0 (N): Maximal force at take-off; V0 (m/s): Maximal velocity of center of mass at take-off; SP 0–10 m (ms): 0–10 m split time; SP 10–20 (ms): 10–20 m split time; SP 0–20 (ms): 0–20 m split time; MCV SQ 80%(m/s): Mean concentric velocity with 80% RM for back squat; MCV SQ 60% (m/s): Mean concentric velocity with 60% RM for back squat; MCV HT 80% (m/s): Mean concentric velocity with 80% RM for hip thrust; MCV HT 60% (m/s): Mean concentric velocity with 60% RM for hip thrust; pow SQ 80% (W): Power in back squat with 80% RM; pow SQ 60% (W): Power in back squat with 60% RM; pow HT 80% (W): Power in hip thrust with 80% RM; pow HT 60% RM (W): Power in hip thrust with 60% RM.

**Table 3:** Effect size of performance variables: Hip thrust group versus back squat group.

Variables	Changes %	Standardized Differences	QI		Chances
–	(90% CL)	(90% CL)	–	–	–
CMJ	−0.5 (−7.1;6.6)	−0.02 (−0.36;0.31)	Small	Unclear	13/69/18
Pow CMJ	−2.5 (−13.7;10.1)	−0.10 (−0.57;0.38)	Trivial	Unclear	14/51/35
F0	−9.8 (−77.3;258.5)	−0.05 (−0.66;0.57)	Trivial	Unclear	24/43/33
V0	−1.4 (−5.0;2.3)	−0.14 (−0.49;0.22)	Trivial	Unclear	6/57/38
SP 0–10m	5.6 (−1.2;12.8)	0.7 (−0.16;1.56)	Medium	Likely	85/11/4
SP10–20m	−0.1 (−4.7;4.3)	−0.02 (−0.8;0.77)	Trivial	Unclear	31/36/34
SP 0–20m	3.1 (−1.3;7.7)	0.46 (−0.2;1.13)	Small	Likely	76/19/5
T-test	1.7 (0.4;3.0)	0.36 (0.08;0.65)	Small	Likely	84/16/0
MCV SQ 80%	−1.0 (21.0;23.9)	−0.03 (−0.7;0.64)	Trivial	Unclear	27/41/32
MCV SQ 60%	−11.7 (−24.4;3.2)	−0.7 (−1.59;0.18)	Medium	Likely	5/12/84
MCV HT 80%	20.0 (−6.8;54.4)	0.53 (−0.2;1.27)	Medium	Likely	78/17/5
MCV HT 60%	22.0 (2.5;45.2)	1.02 (0.13;1.91)	High	Likely	94/5/2
Pow SQ 80%	−5.9 (−24.9;17.8)	−0.17 (−0.80;0.46)	Trivial	Unclear	15/38/47
Pow SQ 60%	−8.2 (−24.6;11.8)	−0.35 (−1.16;0.46)	Small	Unclear	12/25/63
Pow HT 80%	0.4 (−22.1;29.4)	0.01 (−0.55;0.57)	Trivial	Unclear	28/47/26
Pow HT 60%	37.8 (−6.4;103.0)	1.33 (−0.27;2.94)	High	Unclear	89/6/6

QI: Qualitative inferences; CMJ (cm): Countermovement jump; Pow CMJ (w): Power in countermovement jump assessment; F0 (N): Maximal force at take-off; V0 (m/s): Maximal velocity of center of mass at take-off; SP 0–10 m (ms): 0–10 m split time; SP 10–20 (ms): 10–20 m split time; SP 0–20 (ms): 0–20 m split time; MCV SQ 80% (m/s): Mean concentric velocity with 80% RM for back squat; MCV SQ 60% (m/s): Mean concentric velocity with 60% RM for back squat; MCV HT 80% (m/s): Mean concentric velocity with 80% RM for hip thrust; MCV HT 60% (m/s): Mean concentric velocity with 60% RM for hip thrust; pow SQ 80% (W): Power in back squat with 80% RM; pow SQ 60% (W): Power in back squat with 60% RM; pow HT 80% (W): Power in hip thrust with 80% RM; pow HT 60% RM (W): Power in hip thrust with 60% RM.
